# Age-Related Alteration of Arginase Activity Impacts on Severity of Leishmaniasis

**DOI:** 10.1371/journal.pntd.0000235

**Published:** 2008-05-14

**Authors:** Ingrid Müller, Asrat Hailu, Beak-San Choi, Tamrat Abebe, Jose M. Fuentes, Markus Munder, Manuel Modolell, Pascale Kropf

**Affiliations:** 1 Department of Immunology, Faculty of Medicine, Imperial College London, London, United Kingdom; 2 Department of Microbiology, Immunology & Parasitology, Faculty of Medicine, Addis Ababa University, Addis Ababa, Ethiopia; 3 Centro de Investigación Biomédica de Enfermedades Neurodegenerativas (CIBERNED), Departamento de Bioquímica y Biología Molecular, E.U. Enfermería y T.O., Universidad de Extremadura, Cáceres, Spain; 4 Department of Hematology, Oncology, and Rheumatology, University Hospital Heidelberg, Heidelberg, Germany; 5 Department of Cellular Immunology, Max-Planck-Institute for Immunobiology, Freiburg, Germany; Institut Pasteur de Tunis, Tunisia

## Abstract

**Background:**

The leishmaniases are a group of vector-borne parasitic diseases that represent a major international public health problem; they belong to the most neglected tropical diseases and have one of the highest rates of morbidity and mortality. The clinical outcome of infection with *Leishmania* parasites depends on a variety of factors such as parasite species, vector-derived products, genetics, behaviour, and nutrition. The age of the infected individuals also appears to be critical, as a significant proportion of clinical cases occur in children; this age-related higher prevalence of disease is most remarkable in visceral leishmaniasis. The mechanisms resulting in this higher incidence of clinical disease in children are poorly understood. We have recently revealed that sustained arginase activity promotes uncontrolled parasite growth and pathology *in vivo*. Here, we tested the hypothesis that arginase-mediated L-arginine metabolism differs with age.

**Methodology:**

The age distribution of patients with visceral or cutaneous leishmaniasis was determined in cohorts of patients in our clinics in endemic areas in Ethiopia. To exclude factors that are difficult to control in patients, we assessed the impact of ageing on the manifestations of experimental leishmaniasis. We determined parasite burden, T cell responses, and macrophage effector functions in young and aged mice during the course of infection.

**Results:**

Our results show that younger mice develop exacerbated lesion pathology and higher parasite burdens than aged mice. This aggravated disease development in younger individuals does not correlate with a change in T helper cytokine profile. To address the underlying mechanisms responsible for the more severe infections in younger mice, we investigated macrophage effector functions. Our results show that macrophages from younger mice do not have an impaired capacity to kill parasites; however, they express significantly higher levels of arginase 1 than aged mice and promote parasite growth more efficiently. Thus, our results demonstrate that ageing differentially impacts on L-arginine metabolism and subsequent effector functions of physiologically distinct macrophage subsets.

**Conclusions:**

Here, we show that arginase-mediated L-arginine metabolism is modulated with age and affects the capacity of macrophages to express arginase; the increased capacity to upregulate this enzyme in younger individuals results in a more permissive environment for parasite growth, increased disease severity and pathology. These results suggest that the difference in arginase-mediated L-arginine catabolism is likely to be an important factor contributing to the increased incidence of clinical cases in children. Thus, targeting L-arginine metabolism might be a promising therapeutic strategy against leishmaniasis, especially in children and young adults.

## Author Summary

It is well documented that ageing alters many aspects of immune responses; however, a causal relation between impaired immune functions in ageing individuals and the response to infection has not been established. Experimental leishmaniasis is an excellent model to analyse protective and pathological immune responses. *Leishmania* parasites are obligate intracellular pathogens and invade mainly macrophages, which have dual function: they can kill the parasites or promote their growth. We have recently shown that arginase, an enzyme induced in infected macrophages, is a key factor for parasite survival. Here, we show that ageing reduces the expression levels of arginase in macrophages, resulting in more efficient control of parasite growth. Our results suggest that age-related differences in the metabolism of arginase in macrophages might contribute to the higher susceptibility of children to leishmaniasis.

## Introduction

Infections with protozoan parasites inflict an immense toll on the developing world; they are major causes of morbidity and mortality and impede economic development. Leishmaniases are vector-borne diseases, the parasites being transmitted by bites of blood feeding female sandflies and causing different disease manifestations in humans, ranging from the relatively benign self-healing cutaneous form through the disseminated and diffuse cutaneous, to the most severe visceral leishmaniasis. Leishmaniases belong to the most neglected diseases, yet they occur in five continents and are endemic in almost all tropical and subtropical areas [1]. The rising incidence of leishmaniasis around the world is an increasing concern for many countries.

A multitude of factors, including parasite and vector species, host immune responses, genetic and environmental factors influence the outcome of infection. The age of the infected individual also appears to be crucial, as a high proportion of the patients are children. This age-related higher prevalence of disease is most remarkable in visceral leishmaniasis [2],[3],[4],[5],[6],[7],[8],[9],[10]. The mechanisms resulting in this higher incidence of clinical cases in children however are poorly understood.

*Leishmania* are obligate intracellular parasites they survive and replicate predominantly in macrophages. Experimental infections of inbred strains of mice with *Leishmania* (*L.*) *major* have established the current paradigm of T helper (Th) subset involvement in infectious diseases. Control of infection and healing has been associated with a polarized Th1 response whereas non-healing is attributed to a dominant Th2 response [11]. However, the regulation of immune responses against *Leishmania* parasites is complex and Th2 dominance does not fully explain non-healing or reactivated forms of disease [12],[13].

Macrophages, the main effector cells in leishmaniasis, can be instructed to kill or to promote the growth of intracellular *Leishmania* parasites, depending on the balance of two inducible enzymes, nitric oxide synthase 2 (NOS2) and arginase. These two enzymes use a common substrate, L-arginine, and are competitively regulated by type1 and type 2 cytokines [14]. The fate of the intracellular parasite depends on the type of signal the macrophages receive: the type 1 cytokine interferon-γ (IFN-γ) induces classical activation of macrophages and expression of NOS2 that oxidizes L-arginine into nitric oxide (NO), a metabolite responsible for parasite killing [15]; the key type 2 cytokine IL-4 results in alternative activation of macrophages and the induction of arginase [16],[17]. Arginase initiates one of the classic pathways of arginine degradation and it regulates NO synthesis [18]. Two distinct arginase isoforms, encoded by different genes and differing in their cellular localization, have been identified in mammals: type 1 arginase is cytosolic and type 2 arginase is mitochondrial [19]. Arginase hydrolyzes L-arginine to urea and ornithine; the latter being the main intracellular source for synthesis of polyamines necessary for parasite growth. Indeed, we recently showed that sustained arginase activity promotes uncontrolled parasite growth and pathology *in vivo* [20].

In the current study we tested the hypothesis that arginase-mediated L-arginine metabolism is altered with age and thereby modulates the capacity of macrophages to control parasite growth.

## Materials and Methods

### Mice

3–4 and 6–8 week old female BALB/c mice (Charles River, UK) were kept in individually vented cages for up to 18 months. The animal colonies, screened regularly for mouse pathogens, consistently tested negative. Animal experiments were performed in accordance with Home Office and institutional guidelines.

### Patients

Data from 91 patients diagnosed with cutaneous leishmaniasis (CL) at the University of Addis Ababa in the time period from July 2005–April 2007 were used for determination of age distribution of CL. Diagnosis was confirmed by smear and/or NNN culture and later confirmed to be due to *L. aethiopica* by biochemical typing of randomly selected strains. Typing of selected strains was done by a multi-locus enzyme electrophoresis (MLEE) technique at the Istituto Superiore di Sanita in Rome, Italy.

Data from 37 patients diagnosed from 1997–2000 with visceral leishmaniasis (VL) from an endemic region in Ethiopia were used to determine the age distribution of VL. All VL cases were due to *L. donovani* (determined by MLEE) and were diagnosed as described [21]. The presence of parasites in lymph node or splenic aspirates was assessed by direct smear or culture [21].

### Experimental infection with *L. major* parasites

For infections, 2×10^6^ *L. major* LV39 (MRHO/SU/59/P-strain) promastigotes were injected s.c. into the footpad and lesions were monitored as described [22].

### Determination of parasite load

The number of living *L. major* parasites in infected tissues was determined using the parasite limiting dilution assay [22].

### Determination of arginase activity

Arginase activity was measured in macrophage lysates as previously described [14],[20]. One unit of enzyme activity was defined as the amount of enzyme that catalyzes the formation of 1 µmol of urea per min.

Arginase activity at the site of lesions was determined *ex vivo* using 1–10 µl of footpad homogenate, and the method described above.

Amastigotes were purified from the lesions as described in [23] and arginase activity was determined as described above.

### Western Blot Analysis

Arginase 1 protein expression was determined by western blot as described [20], using anti-arginase 1, a rabbit polyclonal antibody raised against rat arginase 1, which cross-reacts with mouse arginase 1 but not arginase 2 [24].

### Cytokine measurements

Draining lymph nodes from individual mice (5×10^6^ cells/ml) were restimulated with 1×10^6^/ml *L. major* promastigotes. Forty-eight hours later, culture supernatants were harvested and cytokines determined by ELISA according to the supplier's protocol (Pharmingen). Detection limits are 20 pg/ml for IL-4, 20 pg/ml for IL-10 and 1 U/ml for IFN-gamma.

The frequency of CD4^+^ T cells expressing IL-4, IL-10 or IFN-γ was determined by flowcytometry as described [25]. Detection of intracellular cytokines was determined using an EPICS XL instrument (Beckman Coulter). Data were analyzed using Beckman Coulter Expo 32 software.

### Proliferation assay

The proliferative capacity of CD4^+^ T cells was determined by flowcytometry, measuring BrdU incorporation as described [26].

### Macrophages

Bone marrow obtained by flushing the femurs of naïve mice was cultured during 8 days in hydrophobic Teflon bags as described [14].

### Macrophage activation markers

Macrophages were stimulated with 100 U/ml IFN-g (Peprotech) and 500 U/ml TNF-α (Peprotech) or with 20 U/ml IL-4 (Peprotech) for 48 hr. BMMΦ (5×10^5^) were incubated with FcR blocking reagent (Pharmingen) for 5 min and anti-CD206 (Serotec), anti-CD40, anti-CD80 or anti-CD86 mAbs (eBioscience) were added for 20 min at 4°C. The expression of CD206, CD40, CD80 and CD86 was determined using an EPICS XL instrument (Beckman Coulter).

### Nitrite determination

NO_2_^−^ accumulation was used as an indicator of NO production and measured using Griess reagent [22].

### Determination of *L. major* parasite growth in macrophages

Mature macrophages (5×10^5^/ml) were plated and stimulated with either 100 U/ml IFN-γ (Peprotech) and 500 U/ml TNF-α (Peprotech) or indicated concentrations of IL-4 or IL-13 (Peprotech) and infected with 25×10^5^/ml *L. major* parasites. After four days the macrophages were washed, lysed and the number of viable parasites was determined as described [22].

### Statistical analyses

Statistical differences were determined using a two-tailed Mann-Whitney test. Differences were considered statistically significant at *P*<0.05.

## Results

### Effect of age on disease development

**Figure 1:**
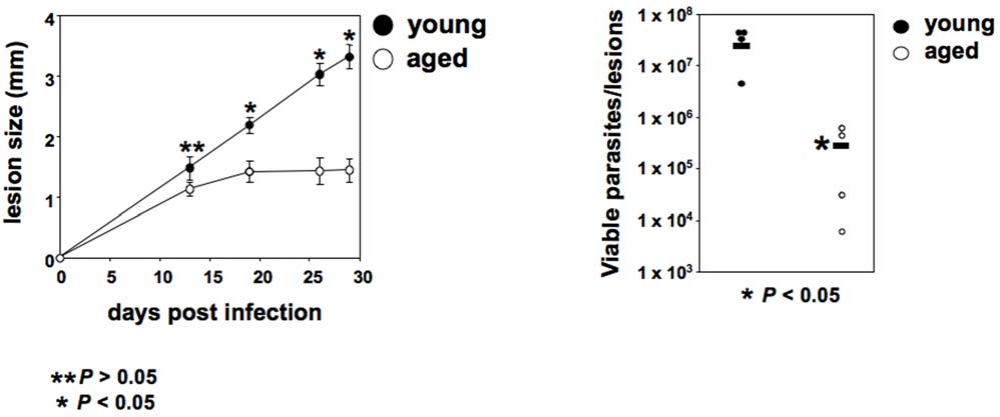
Severity of experimental leishmaniasis is increased in young BALB/c mice. Groups of young (6–8 weeks) and older (12 months) BALB/c mice were infected with 2×10^6^ *L. major* promastigotes in one hind footpad. (a) The lesion development was monitored by measuring the increase in footpad thickness at regular intervals; the error bars represent standard error of the mean; (b) the parasite load was determined by limiting dilution assay 4 weeks post infection; the horizontal bar represents the average value. Data show the results of one representative experiment out of six independent experiments.

To assess the impact of ageing on the development of experimental leishmaniasis, genetically susceptible young (6–8 week) and older (12 months) BALB/c mice were infected s.c. with *L. major* parasites and lesion development and parasite load were determined. The onset of lesion development was similar in both groups of mice. After three weeks of infection, the young BALB/c mice started to develop ulcers and 4 weeks later, the experiment had to be terminated owing to severity of the lesions. In contrast, older BALB/c mice had clearly reduced pathology; they did not develop ulcers and their lesions stabilized (Figure 1a). Consistent with the exacerbated pathology and increased lesion size, the parasite load at the site of the lesion was significantly higher in the young than in old BALB/c mice (Figure 1b). Similar results were obtained with mice aged 8, 12 and 18 months (data not illustrated). These results show that disease pathology is clearly exacerbated in young BALB/c mice.

**Figure 2:**
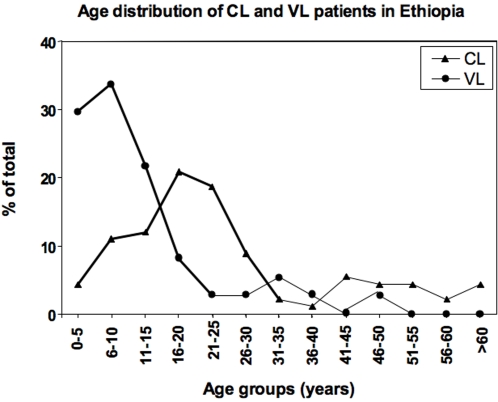
Age distribution of visceral and cutaneous leishmaniasis. The age distribution of patients suffering from visceral (n = 37, circle) or cutaneous (n = 91, triangle) leishmaniasis was determined in endemic areas of Ethiopia.

Data obtained on leishmaniasis in our clinics in endemic regions of Ethiopia show that 67% of cases of cutaneous leishmaniasis occur in people younger than 26, and even more striking, 85% of cases of visceral leishmaniasis occur in children under 16 years (Figure 2). Thus in the human, the majority of the clinical cases appears to occur in younger individuals. Whereas many factors are likely to contribute to this higher prevalence of clinical cases in younger individuals, these data supports our finding that age is a major factor in the control of disease.

### Increased disease severity is not associated with a significant alteration of the Th phenotype

**Figure 3:**
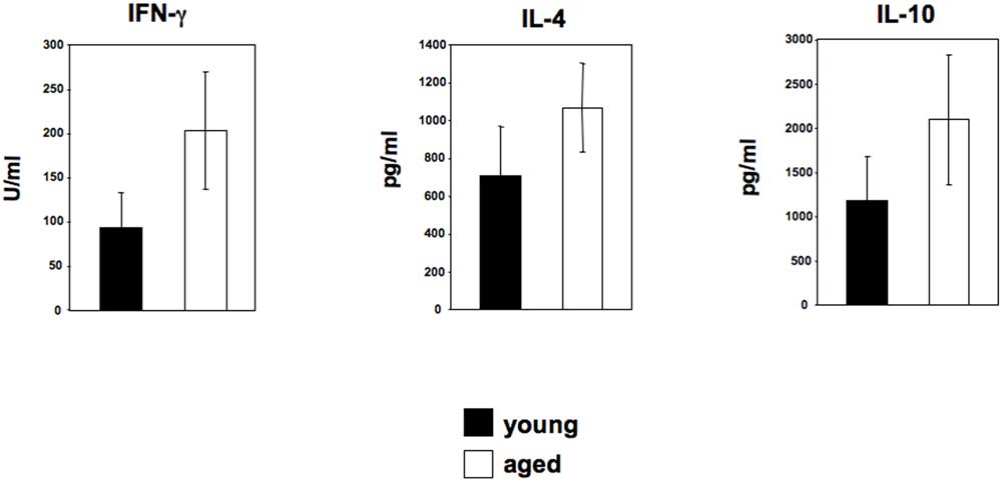
Exacerbation of disease in young BALB/c mice is not associated to a switch in Th phenotype. Groups of young (6–8 weeks) and older (12 months) BALB/c mice were infected with 2×10^6^ *L. major* promastigotes in one hind footpad. Four weeks post infection, the popiteal lymph nodes were harvested and restimulated with *L. major* parasites. The supernatant were harvested and tested for their content of cytokines by ELISA. The error bars represent standard error of the mean and data show the results of one representative experiment out of three independent experiments.

To assess whether the increased disease severity (Figures 1a and b) observed in young BALB/c mice correlated with modulation of the T helper phenotype, we determined antigen-specific cytokine production by lymph node cells. Interestingly in all experiments performed there was a consistent increase in the levels of antigen-specific IFN-γ, IL-4 and IL-10 in the aged mice, however, these differences were not statistically significant (P>0.05) (Figure 3 and Table 1). In agreement with these results, the frequencies of CD4^+^ T cells expressing IFN-γ, IL-4 or IL-10 were not significantly different between young and aged mice (Table 2). In addition, the capacity of CD4^+^ T cells to proliferate in response to *L. major* parasites was similar (Table 2).

**Table 1:** Cytokine production.

Exp. 1	IFN-γ (U/ml)	IL-4 (pg/ml)	IL-10 (pg/ml)
**Young**	92.8±39.4	703.5±261.5	1181.8±499.0
**Aged**	202.8±66.8	1066.0±232.7	2092±733
Exp. 2			
**Young**	33.7±8.8	1234.7±212.4	1650.8±202.3
**Aged**	86.9±30.9	1579.7±261.1	2204±594.4
Exp. 3			
**Young**	28.0±7.4	899.5±144.0	1425.3±360.7
**Aged**	50.3±10.4	1033.5±76.1	1723.3±276.7

Groups of young (6–8 weeks) and older (12 months) BALB/c mice were infected with 2×10^6^ *L. major* promastigotes in one hind footpad. Four weeks post infection, the popliteal lymph nodes were harvested and restimulated with *L. major* parasites. The supernatant were harvested and tested for their content of cytokines by ELISA. The data represent the cytokine concentration±standard error of the mean and data show the results of three independent experiments are represented.

**Table 2:** Proliferation and frequency of cytokine-producing CD4^+^ T cells.

	Proliferation	Cytokine expression		
	% CD4^+^BrdU^+^	% CD4^+^IFN-γ^ +^	% CD4^+^IL-4^+^	% CD4^+^IL-10^+^
**Young**	28.7±3.7	16.4±2.6	16.2±3.8	11.0±2.5
**Aged**	28.0±8.5	20.4±3.3	15.0±3.4	10.0±1.1

Groups of young (6–8 weeks) and older (12 months) BALB/c mice were infected with 2×10^6^ *L. major* promastigotes in one hind footpad. Four weeks post infection, the popiteal lymph nodes were harvested and restimulated with *L. major* parasites. The frequency of cytokine-expressing CD4^+^ T cells was determined by intracellular cytokine staining and the frequency of proliferating CD4^+^ T cells was determined by BrdU incorporation.

Data show the results of one representative experiment out of three independent experiments.

These results indicate that increased disease severity in young BALB/c mice is unlikely to be due to alterations in the key Th cytokine profile.

### Higher arginase activity at the site of pathology correlates with disease exacerbation

**Figure 4:**
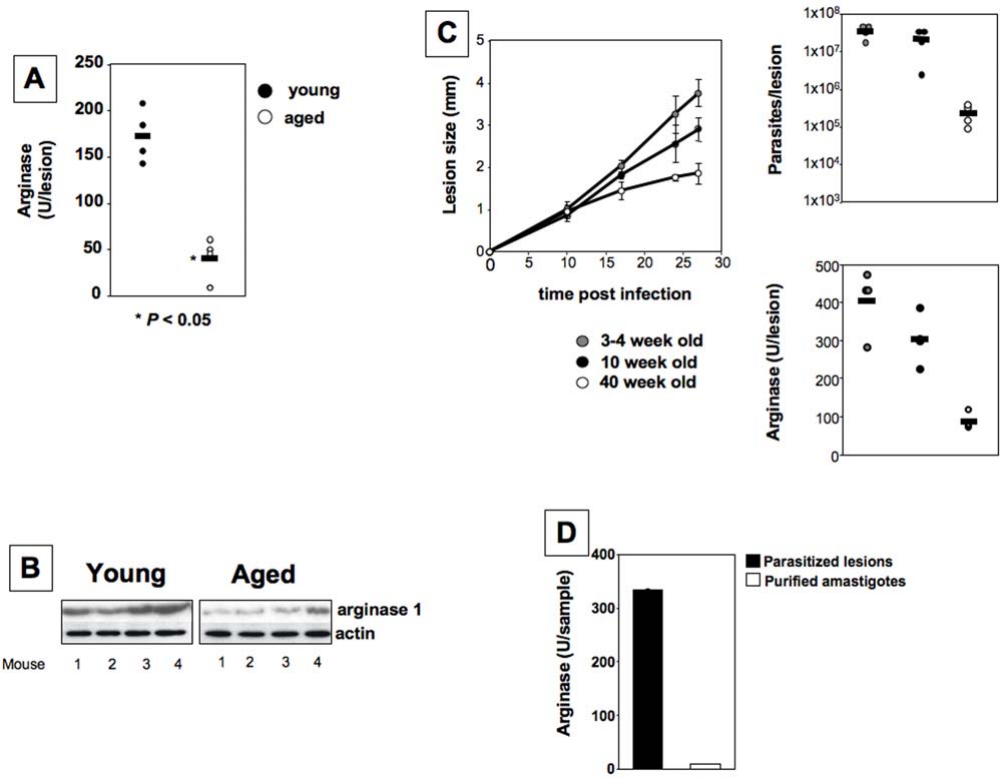
Higher arginase activity at the site of pathology correlates with exacerbation of disease. Groups of young (6–8 weeks) and older (12 months) BALB/c mice were infected with 2×10^6^ *L. major* promastigotes in one hind footpad. Four weeks post infection, the infected footpads were harvested and the level of arginase activity was determined by enzymatic assay (a) or western blot (b). (c) Groups of pre-pubertal mice (3–4 weeks), adult (10 weeks) or older (40 weeks) BALB/c mice were infected with 2×10^6^ *L. major* promastigotes in one hind footpad and the lesion development was monitored by measuring the increase in footpad thickness at regular intervals; the error bars represent standard error of the mean (left panel); 4 weeks post infection, the infected footpads were harvested and the parasite load (upper right panel) and arginase activity (lower right panel) were measured. (d) BALB/c mice were infected with 2×10^6^ *L. major* promastigotes in one hind footpad. Four weeks post infection, the infected footpads were harvested and arginase activity was determined both in the footpads and in the amastigotes purified from the footpads. The horizontal bar represents the average value and data show the results of one representative experiment out of three independent experiments.

We have recently shown that in *L. major* infected healer strains of mice (CBA, C57BL/6), arginase activity is low, only detectable during active disease. In contrast, increasing arginase activity was detected at the local site of lesion, and clearly correlated with uncontrolled parasite replication and lesion size in nonhealing BALB/c mice [20]. To assess whether the exacerbation of disease in young mice was also associated with higher arginase expression at the site of infection, we determined the activity of this enzyme. The data presented in Figure 4a do indeed show significantly higher arginase activity in the lesions of young BALB/c mice than in those of their older counterparts. We also determined arginase 1 protein expression by Western blot and found arginase to be higher in the lesions of young mice (Figure 4b).

Hormones such as estrogens can influence resistance to infections [27],[28],[29],[30],[31]; since estrogens are significantly higher in mice of reproductive age, in the next experiment we use pre-pubertal mice aged 3–4 weeks and determined whether they displayed an altered resistance to *L. major* infection. As shown in Figure 4c, the lesion size was slightly higher in 3–4 weeks old mice as compared to those of young adult mice (10 week old). Importantly lesion sizes from 3–4 and 10 week old mice were significantly higher then those of aged mice (40 week old). Accordingly, both parasite loads and arginase activities at the site of infection were significantly increased in both pre-pubertal and adult mice as compared to aged mice.

The increased arginase activity and arginase protein expression in the lesions of young *L. major* infected BALB/c mice support our hypothesis that arginase mediated L-arginine metabolism alters with age. These results further confirm our previous conclusion that the level of arginase activity correlates with disease severity [20].

We and others have shown that *Leishmania* parasites express their own arginase [20],[32],[33]. Therefore, to measure the contribution of parasite arginase to the total levels of arginase activity measured in the lesions of BALB/c mice, we measured arginase in the whole lesions, as well as in amastigotes purified from lesions. As shown in Figure 4d, we could detect low levels of arginase activity in the purified amastigotes, however it was 40.5-fold lower as compared to that detected in the parasitised lesions. No arginase 1 protein was detectable by Western blot in the purified amastigotes (data not shown). These results show that arginase activity from amastigotes only contributes minimally to arginase activity measured in the lesions.

### Ageing does not affect classically activated macrophages

**Figure 5:**
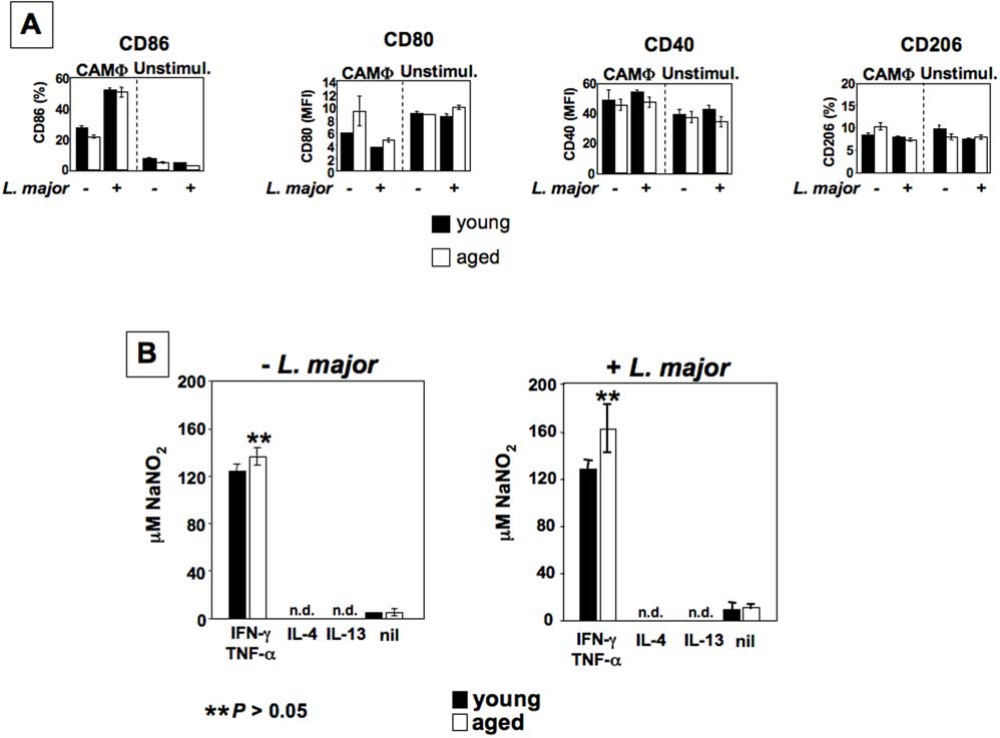
Classically activated macrophages are not affected by ageing. BMMΦ were derived from young (6–8 weeks) and older (12 months) BALB/c mice and stimulated into CAMΦ with IFN-γ and TNF-α or into AAMΦ with IL-4 or IL-13. (a) Activation markers were determined by flow cytometry; (b) NO production was assessed by using the Griess reagents. The error bars represent standard deviation and data show the results of one representative experiment out of four independent experiments.

Since macrophages are the ultimate effector cells, and arginase activity is increased in lesions of young mice, we reasoned that macrophages derived from old and young mice must differ in the efficiency of their effector functions. To test this hypothesis, we first examined the expression of activation markers on classically activated macrophages (CAMΦ) No differences were detected in the expression of CD86, CD40, CD80 and CD206 between CAMΦ from young and older mice (Figure 5a). We also assessed the capacity of CAMΦ from young and old mice to upregulate NOS2 and produce NO. MΦ from both groups produced similar levels of NO in response to IFN-γ/TNF-α in the absence or presence of parasites (Figure 5b). Furthermore, we determined NO production by CAMΦ derived from pre-pubertal, adult and aged mice. As shown in Table 3, NO production was similar in all age groups.

**Table 3:** NO_−_ production by activated macrophages.

Age groups	+*L. major*	−*L. major*
**3–4 weeks**	70.9±3.3	65.6±1.2
**10 weeks**	67.6±6.1	62.5±0.3
**40 weeks**	71.8±0.1	67.5±0.5

BMMΦ were derived from pre-pubertal (3–4 weeks), young adult (10 weeks) and older (40 weeks) BALB/c mice and differentiated into CAMΦ with IFN-γ and TNF-α and NO_−_ production was assessed by using the Griess reagents.

Data show the results of one representative experiment out of two independent experiments.

We conclude that ageing does not affect the capacity of CAMΦ to induce NOS2 or to generate NO and did not alter expression levels of activation markers in CAMΦ.

### Alternatively activated macrophages derived from young mice express higher levels of arginase

**Figure 6:**
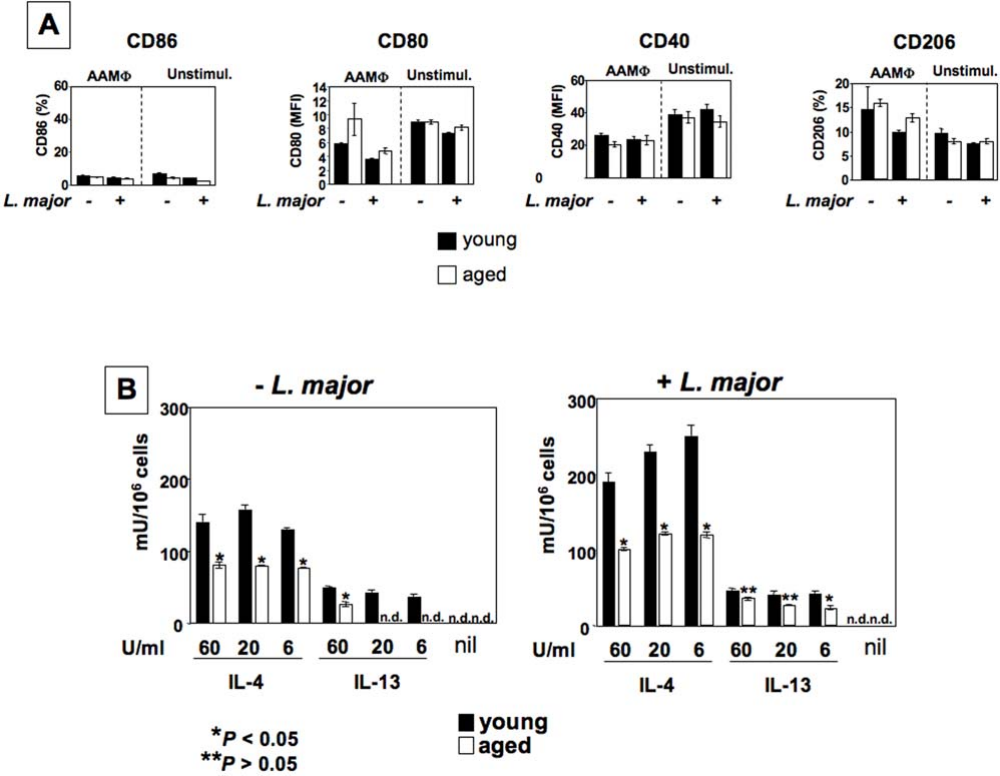
Arginase expression is increased in alternatively activated macrophages from young mice. BMMΦ were derived from young (6–8 weeks) and older (12 months) BALB/c mice and differentiated into AAMΦ with IL-4 or IL-13. (a) Activation markers were determined in *L. major* infected and uninfected cells by flow cytometry; (b) arginase activity was measured by enzymatic assay. The error bars represent standard deviation and data show the results of one representative experiment out of four independent experiments.

We next examined the capacity of alternatively activated (AA) MΦ from young and old mice to exert their effector functions. AAMΦ were obtained by differentiating mature bone marrow MΦ with IL-4 or IL-13 alone, as IL-10, which synergizes with IL-4 and strongly enhances the expression levels of arginase, acts on a different receptor [17]. We found no differences in the expression of CD206, CD40, CD80 and CD86 between AAMΦ derived from young and older mice (Figure 6a). We then assessed the capacity of macrophages from young and older mice to induce arginase in response to IL-4 or IL-13. As shown in Figure 6b, upregulation of arginase was observed in both age groups of AAMΦ, and infection with *L. major* parasites enhanced the arginase levels even further. Importantly, AAMΦ derived from young mice displayed significantly higher arginase activity in response to IL-4 or IL-13. We also examined the capacity of AAMΦ derived from pre-pubertal BALB/c mice (3–4 weeks) to express arginase; as shown in Table 4, similarly to adult mice, they express significantly more arginase then aged mice (40 weeks). These results show that ageing alters the capacity of AAMΦ to express arginase.

**Table 4:** Arginase activity in activated macrophages.

Age groups	+*L. major*	−*L. major*
**3–4 weeks**	266.7±23.6	243.1±33.3
**10 weeks**	232.7±23.8	221.1±50.3
**40 weeks**	155.6±21.3	135.2±23.8

BMMΦ were derived from pre-pubertal (3–4 weeks), adult (10 weeks) and older (40 weeks) BALB/c mice and differentiated into AAMΦ with 20 U/ml IL-4 and arginase activity was measured by enzymatic assay.

Data show the results of one representative experiment out of two independent experiments.

### Higher arginase activity correlates with increased parasite replication in macrophages derived from young mice

The polyamines resulting from the hydrolysis of L-arginine by arginase are essential for parasite growth [20],[34]. Here we hypothesize that lower arginase activity in AAMΦ from older mice (Figures 4a, b and 6b) is responsible for the decrease in parasite replication.

**Figure 7:**
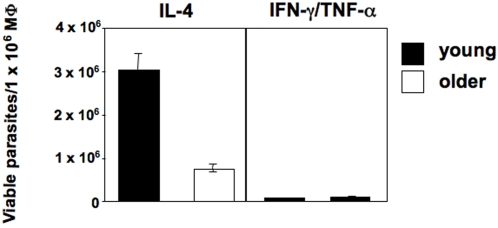
Alternatively activated macrophages from young mice have an increased capacity to promote parasite growth. BMMΦ were derived from young (6–8 weeks) and older (12 months) BALB/c mice, differentiated into CAMΦ with IFN-γ and TNF-α or into AAMΦ with IL-4 and infected with *L. major*. Four days later, the number of viable parasite per 1×10^6^ MΦ was determined by LDA.

To determine whether AAMΦ from young and old mice support *L. major* replication differently, we examined the number of viable parasites by limiting dilution assay. There is a clearly higher parasite load in AAMΦ derived from young mice compared to those in older mice (Figure 7), indicating that AAMΦ from young mice promote parasite growth more effectively then AAMΦ from older mice. CAMΦ from both groups, however, are equally efficient in parasite killing (Figure 7), suggesting transport of L-arginine is sufficient in both age groups.

The results show that AAMΦ from young mice provide a more permissive environment for parasite growth, directly linking the higher arginase activity (Figures 1b, 4 and 6) to increased parasite replication (Figure 7).

## Discussion

Our results show that younger BALB/c mice develop clearly exacerbated disease as compared to older mice, as demonstrated by more pronounced lesions, pathology and higher parasite burdens at the site of parasite inoculation. This aggravation of disease correlates with a higher arginase activity expressed at the site of infection. This is due to altered macrophage effector functions, as AAMΦ derived from younger mice have an increased capacity to express arginase. To our knowledge, this is the first report showing an age-related modulation of arginase expression. Importantly, arginase activity *in vivo* was also increased at the site of lesion in *L. major* infected younger BALB/c mice. We have recently shown that sustained arginase activity promotes uncontrolled parasite growth in BALB/c mice and is pivotal for parasite growth [20]. Thus, increased arginase activity in macrophages from young mice results in increased L-arginine catabolism and consequently increased levels of polyamines, providing a more permissive environment for parasite replication.

The reduced parasite growth in macrophages from elderly mice could be due to enhanced parasite killing or reduced parasite growth or a combination of both. The results presented in Fig. 5b show that the potential of bone marrow derived classically activated macrophages to generate nitric oxide is comparable in cells derived from young, adult and elderly mice.

In contrast, the potential to induce arginase activity declined with age and the presence of *L. major* parasites further enhanced arginase activity (Fig. 6b). Both arginase and iNOS compete for utilizing L-arginine, however, this does not necessarily indicate that the increased susceptibility is due insufficient parasite killing. Improved control of parasite growth in the elderly is not necessarily due to a shift of the L-arginine metabolism towards the iNOS pathway and enhanced NO production. The expression of both of the L-arginine metabolizing enzymes in macrophages is induced by type 1 and type 2 cytokines, respectively and activation of macrophages results in increased expression of cationic amino acid transporters and increased transport of L-arginine into the macrophages; both enzyme levels and L-arginine transport determine the catabolic rate. Thus, the net-effect of unaltered generation of NO ( = constant rate of killing) and reduced induction of arginase activity ( = reduced nutrients, reduced growth) with age is likely to account for the improved disease control. Indeed, we previously showed that arginase directly regulates parasite growth without affecting NO synthesis [20]. In this context, it is important to note that macrophages synthesize proline via the arginase pathway and this macrophage-generated proline could also be used by the parasites. In agreement with our findings, arginase 1 has been shown to be limiting for polyamine synthesis without affecting NO levels in activated macrophages [35],[36].

We have previously shown that the levels of arginase 1 expressed by *L. major* parasites influence their virulence [32] and parasite arginase can catabolize radioactive labelled L-arginine [32]. Thus, parasite arginase activity is likely to influence the early immune response. Importantly, the levels of arginase expressed by intracellular *L. major* amastigotes are negligible as compared to macrophage arginase (Figure 4d) [32]. In agreement with these findings, a recent publication showed that arginase activity expressed by *L. mexicana* amastigotes is only a minute fraction compared to the macrophages arginase activity (0.418 in macrophages, 0.0235 by parasites [37]).

In conclusion, these *in vitro* results indicate that macrophages from aged mice provide a less favourable environment for parasite growth than those from younger mice due to reduced nutrient availability.

Ageing is generally associated with a decline of immune responses [38] and some responses can be improved by targeting L-arginine metabolism [39]. L-arginine administration is beneficial in conditions such as trauma, stress, burn or injury, it improves immune functions and facilitates wound healing [40].

Arginase plays a crucial role in wound healing, a process severely impaired in the elderly. During wound healing, NOS and arginase are induced in a time-coordinated manner [41], with NOS expression peaking first and to be particularly important in initiating the inflammatory phase. Arginase is expressed later and is of prime importance in actual wound healing; the conversion of L-arginine into urea and ornithine and its metabolism to polyamines initiates the repair phase of the inflammatory response [42]. The reduced expression of arginase in macrophages from older individuals is likely to result in lower levels of ornithine, thereby reducing collagen formation and the proliferation of cells, which might then result in delayed or ineffective wound healing.

In experimental leishmaniasis, uncontrolled disease development is generally associated with a strong Th2-type response [11], however, strong polarized Th2 responses are not sufficient to explain fully nonhealing [12],[13]. In agreement with the cited studies, our results show that amelioration of disease in older mice does not depend on a switch from a Th2- to a Th1-type response. The analysis of the Th1-Th2 cytokine balance during ageing has often resulted in contradictions; both impaired [43] and enhanced Th2 responses [44] have been reported. Factors such as the use of different strains of mice and parasites are likely to contribute to contradictory results.

In support of the increased incidence of leishmaniasis in children and young adults two studies, using mathematical models, have shown an association between age and rate of *Leishmania* infection and suggest that susceptibility to disease declines with age [45],[46]. However, the relationship between age and susceptibility to disease remains an open question. The development and manifestation of disease is the result of a dynamic interplay of a range of different environmental, host-, vector- and parasite-derived factors. Many factors such as nutrition, co-infections, behaviour, differences in metabolic pathways, and prolonged exposure to repeated bites by infected sandflies are important, but difficult to measure in patients. It still remains unclear why there are fewer clinical cases in older people; acquired immunity to re-infection is likely to be on of the main factors; indeed, unexposed adults display a higher susceptibility to infection as compared to the population living in endemic areas [47]. Furthermore, asymptomatic infections appear to provide natural immunisation [48]. In another study where VL was shown to be a disease of children, it was shown that there was a significantly higher prevalence of adults with positive Leishmanin Skin Test (LST), suggesting that they had acquired immunity [49] However, children remain the main risk group for VL [46] and in several outbreaks, children were the population most at risk [49],[50],[51],[52],[53] suggesting that acquired immunity alone might not be sufficient to explain age related higher prevalence of leishmaniasis. The higher level of protection against leishmaniasis with age could also be due to qualitative differences in cell-mediated immune responses in children and older people.

Based on our results, we propose that increased arginase expression and L-arginine catabolism contribute to exacerbation of leishmaniasis in younger individuals. The therapeutic regulation of L-arginine metabolism may provide a target for disease intervention.
